# Retraction: New Diagnosis and Therapy Model for Ischemic-Type Biliary Lesions following Liver Transplantation—A Retrospective Cohort Study

**DOI:** 10.1371/journal.pone.0222409

**Published:** 2019-09-06

**Authors:** The PLOS ONE Editors

Concerns have been raised that the transplants performed in the local context at the time of procedures reported in this article [1] may have involved organs/tissues procured from prisoners [2].

Details as to the donor sources and methods of obtaining informed consent from donors were not reported in [1], and the authors did not clarify these issues or the cause(s) of donor death in response to journal queries. International ethics standards call for transparency in organ donor and transplantation programs and clear informed consent procedures including considerations to ensure that donors are not subject to coercion.

In addition, the authors did not provide documentation when requested by the journal to confirm that the study had institutional ethics approval, and they did not respond to inquiries about the availability of underlying data supporting this study.

Owing to the lack of documentation to demonstrate this study had prospective ethical approval, insufficient reporting, unresolved concerns around the source of transplanted organs and whether they included organs from prisoners, and in compliance with international ethical standards for organ/tissue donation and transplantation, the *PLOS ONE* Editors retract this article.

The first and third author notified the journal that all authors agree with the retraction. The other authors either could not be reached or did not respond directly.

## References

[1] Zhang Y-c, Qu E-z, Ren J, Zhang Q, Zheng R-q, Yang Y, et al (2014) New Diagnosis and Therapy Model for Ischemic-Type Biliary Lesions following Liver Transplantation—A Retrospective Cohort Study. PLoS ONE 9(9): e105795 10.1371/journal.pone.0105795 25192214 PMC4156319

[2] Rogers W, Robertson MP, Ballantyne A, Blakely B, Catsanos R, Clay-Williams R, et al Compliance with ethical standards in the reporting of donor sources and ethics review in peer-reviewed publications involving organ transplantation in China: a scoping review. BMJ Open 2019;9(2): e024473 10.1136/bmjopen-2018-024473 30723071 PMC6377532

